# Case Report: ALPPS hepatectomy, an alternative to liver transplantation in central PRETEXT III hepatoblastomas: a case series

**DOI:** 10.3389/fped.2024.1350697

**Published:** 2024-03-20

**Authors:** Isabel Cristina Garcia Moreno, Sergio Alzate-Ricaurte, Edgar Dario Alzate Gallego, Daniela Hincapie-Ayala, Oscar Javier Serrano Ardila, Jorge Ivan Villegas Otalora

**Affiliations:** ^1^ Department of Pediatric Surgery, Fundación Valle del Lili, Cali, Colombia; ^2^ School of Medicine, Universidad ICESI, Cali, Colombia; ^3^ Centro de Investigaciones Clinicas, Fundación Valle del Lili, Cali, Colombia; ^4^ Department of Transplant Surgery, Fundación Valle del Lili, Cali, Colombia

**Keywords:** hepatoblastoma, ALPPS, PRETEXT III, liver transplantation, case series

## Abstract

**Introduction:**

Hepatoblastoma is the most common malignant primary liver tumor in the pediatric population, accounting for 67% of cases in the United States. Surgical resection is the only curative treatment option; however, it can be performed in only 10% of patients with primary tumors. The two most common limitations for resection are the need for extensive resections and tumors in central locations. The therapeutic hypertrophy of healthy tissue achieved with ALPPS (Associating Liver Partition and Portal vein ligation for Staged Hepatectomy) enables larger resections and has been successfully employed in the pediatric population in recent years.

**Objective:**

To present three cases of patients with centrally located PRETEXT II or III hepatoblastomas who underwent ALPPS procedure as a viable therapeutic alternative to liver transplantation.

**Discussion and results:**

Central PRETEXT III hepatoblastomas are typically indications for liver transplantation. Transplantation offers high five-year survival rates (73%). However, the associated morbidity, healthcare system costs, and limited availability make it necessary to explore alternative options. Series have reported the successful application of the ALPPS procedure in PRETEXT II and PRETEXT III hepatoblastomas in other locations. Therapeutically induced hypertrophy, characterized by an increase in the volume of healthy tissue in unaffected lobes or segments, enabled the resection of previously deemed unresectable lesions. The patients experienced uncomplicated postoperative courses and expected reduction in tumor markers. Chemotherapy selection followed the guidelines outlined in Block C of the SIOPEL IV protocol.

**Conclusions:**

ALPPS hepatectomy is a viable therapeutic option for patients with centrally located PRETEXT III or II hepatoblastomas.

## 1 Introduction

Hepatoblastoma is the most common primary malignant liver tumor in the pediatric population, accounting for 67% of cases in the United States (1). Cases are more frequently observed in the first three years of life, and there has been an increase in disease prevalence, which is attributed to improved survival rates over the last three decades (30%–80%) (2). The use of cisplatin-based chemotherapy has enabled 50%–85% of tumors previously considered unresectable to become resectable (2). However, curability remains primarily centered around radical surgical resection, which can only be performed in 10% of patients with primary tumors (2, 3). This limitation is principally due to inadequate future liver remnants for extensive hepatic resections (3).

Central location PRETEXT II or III hepatoblastomas are indicative of liver transplantation due to their challenging resectability (4). The ALPPS (Associating Liver Partition and Portal vein ligation for Staged Hepatectomy) hepatectomy technique, initially described by Schlitt et al. in adults in 2012 (5), has recently been successfully adopted for the management of pediatric patients. Its use has been described in PRETEXT II and PRETEXT III hepatoblastomas of non-central locations (3, 5–7). Three cases of central location PRETEXT II or III hepatoblastomas in which the ALPPS hepatectomy was successfully employed as an alternative to liver transplantation are being reported for the first time.

## 2 Case 1

A 4-month-old male patient, with no prior medical history, was admitted due to abdominal distension. An MRI was performed, revealing a PRETEXT III hepatoblastoma of a central location, with significant involvement of the right hepatic lobe. Additionally, an initial alpha-fetoprotein level was reported as exceeding 1,000 ng/ml. A liver biopsy diagnosed an epithelial-type hepatoblastoma with a fetal pattern. Initial staging did not reveal any metastatic involvement, so chemotherapy was initiated using the SIOPEL 3 protocol. The patient received a total of five cycles of chemotherapy with Cisplatin. Following the completion of neoadjuvant therapy, follow-up imaging showed a reduction in the size of the mass, being classified as POSTTEXT III with no involvement of vascular structures. As a result, the patient was referred for interdisciplinary management at a higher complexity institution.

Upon admission, physical examination revealed a liver palpable up to 4 cm below the costal margin, extending to the midline of the abdomen, with no other clinical findings or symptoms. Serum alpha-fetoprotein levels were measured at 388.66 ng/ml. To characterize the liver lesions, a three-phase liver and bile duct angiotomography was performed, which showed a liver lesion measuring 6.6 × 5.3 × 8.0 cm in segments IVb and V. This lesion displaced the gallbladder in a posterior-inferior and medial direction and pushed the right hepatic artery, right portal vein, and right suprahepatic vein posteriorly without compromising their caliber. The total hepatic volume was calculated to be 329.7 cc. The FLR-V, corresponding to the theoretical remaining segments II and III, was calculated to be 26% of the total, amounting to 85.67 cc. Preoperative liver function tests revealed a mild increase in AST (Aspartate Aminotransferase) (40.8 U/L, reference value: 0–32), with no additional abnormalities. There were no signs of extrahepatic infiltration. Additionally, a chest CT did not reveal any metastatic lesions to the lungs.

**Figure 1:**
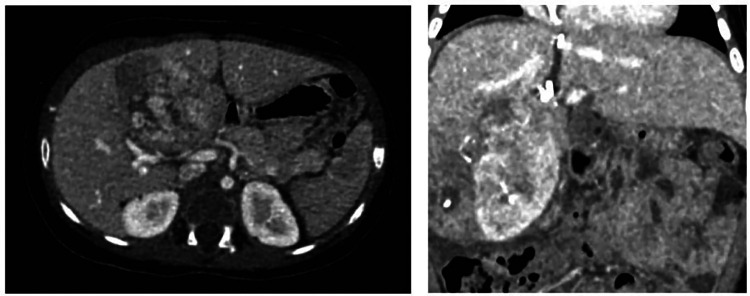
Three-phase liver angiotomography evaluation of Case 1. (Left) Preoperative. (Right) Hypertrophy and surgical changes following stage 1 of ALPPS procedure.

Based on this, the patient's case was reviewed in a multidisciplinary meeting with the Pediatric Oncology/Hematology, Pediatric Transplant Surgery, and Pediatric Hepatology departments, where performing an ALPPS hepatectomy was determined to be the best option. This decision was made to reduce the risk of liver failure. It was based on the calculation that during surgery, a larger resection might be necessary, that could result in a remaining liver volume below the accepted safe thresholds and to avoid liver transplantation. The surgeries were performed by a general surgery specialist, who has completed fellowships in both pediatric surgery and transplant surgery, and assisted by an additional transplant surgeon. During the first surgical procedure, an 8 cm mass was found, compromising segments IV and V in proximity to the right portal pedicles and the umbilical fissure. The first stage of the ALPPS procedure involved ligating the right portal vein and its branches in segment IV, promoting hypertrophy of the left lobe. Hepatic parenchymal transection was also performed. In the immediate postoperative period, the patient was transferred to the pediatric ICU without experiencing any postoperative complications. On the ninth postoperative day, a new three-phase liver and bile duct angiotomography was performed, revealing the expected hypertrophy of the left hepatic lobe, with an increase in volume from 85 cc to 155 cc (Figure 1). On the 12th postoperative day, the patient underwent a second surgical procedure, during which an extended right hepatectomy was performed, involving segments V, VI, VII, VIII, IVa, and IVb. In the immediate postoperative period, there was an elevation in alpha-fetoprotein levels, reaching a value of 560.75 ng/dl, which was attributed to the surgical intervention, with a subsequent progressive decline.

The pathological examination of the specimen revealed a hepatoblastoma with mixed mesenchymal and epithelial differentiation (70% and 30%, respectively). Despite a partial response to chemotherapy, evidenced by 10% necrosis, microcalcifications, venous thrombosis, and histiocytic reaction, tumor viability remained high at 90%. There was no vascular invasion, and the resection margin was clear at 7 mm. Given the tumor's poor response to the initial chemotherapy regimen, as discussed at the tumor committee, and in alignment with the pathology findings, it was decided to switch to the SIOPEL 4 protocol. This decision included post-operative chemotherapy consolidation using Block C, which includes carboplatin and doxorubicin, as indicated for cases showing a suboptimal response to initial treatment. The first cycle of chemotherapy was administered during the hospital stay, and the patient was discharged on the 8th day following the second surgical procedure. The patient was later readmitted for the administration of the remaining chemotherapy cycles.

At the 1 year post-surgical follow-up, no abnormalities were detected on physical examination. The patient has shown a gradual decrease in alpha-fetoprotein (AFP) levels, measured monthly, and there is no evidence of recurrence in either the chest x-ray or abdominal ultrasound, which has been performed quarterly.

## 3 Case 2

A 3-year-old female patient, who had no prior medical history and who resided in a rural area with limited access to healthcare, was admitted to a hospital in the nearest large city due to the presence of an abdominal mass. An ultrasound revealed the presence of a solid, heterogeneous image measuring 10 × 5.7 × 6.3 cm, with an approximate volume of 200 cc. AFP levels were measured at 4,680 ng/ml. Further imaging was conducted, including an abdominal MRI, which reported a multilobulated mass involving segments VII, VIII, VI, and V, measuring 12 × 11 × 6.5 cm, suggestive of a PRETEXT II hepatoblastoma, with no evidence of extrahepatic involvement. Staging was completed with a chest CT, which showed a 2 mm left apical pulmonary micronodule, likely reactive. A biopsy of the lesion was taken, revealing an hepatoblastoma with 90% fetal and epithelial differentiation and 10% embryonic differentiation. Additionally, as part of the initial evaluation studies, a diagnosis of toxoplasmosis without systemic involvement was made. Management with trimethoprim-sulfamethoxazole was prescribed for a duration of 6 weeks. Based on these studies, neoadjuvant management was initiated following the PLADO protocol (based on SIOPEL 3). However, during this course of treatment, the patient developed sepsis associated with a peripherally inserted central catheter, caused by Acinetobacter Baumannii and antibiotic treatment with cefepime was administered. The patient was referred to a higher complexity institution to consider the possibility of a liver transplant.

Upon admission to this institution, the case was discussed in a multidisciplinary meeting involving the Pediatric Oncology/Hematology, Pediatric Transplant Surgery, and Pediatric Hepatology departments. It was decided to continue chemotherapy according to the SIOPEL 4 protocol, specifically cycles A2 and A3, and then perform an evaluation with imaging upon completion. During this period, the patient contracted COVID-19; however, did not develop complications. A three-phase liver angiotomography performed after completing neoadjuvant therapy revealed the presence of a solid mass with lobulated margins and heterogeneous enhancement, measuring 7 × 5 cm (previously 9 cm) in segments VII and VIII. It was in contact with the right lateral side of the middle hepatic vein, with no evidence of extrahepatic disease. The total hepatic volume was calculated to be 597 cc. The future liver remnant volume (FLR-V), corresponding to the theoretical remaining segments II and III, was calculated to be 18% of the total, amounting to 108 cc. Segments for the future liver remnant volume (FLR-V) calculations were chosen in anticipation of an extended right hepatectomy. This surgical decision was driven by the need to address venous drainage issues, necessitating the ligation of the suprahepatic middle and right veins. Preoperative liver function tests revealed no abnormalities.

**Figure 2:**
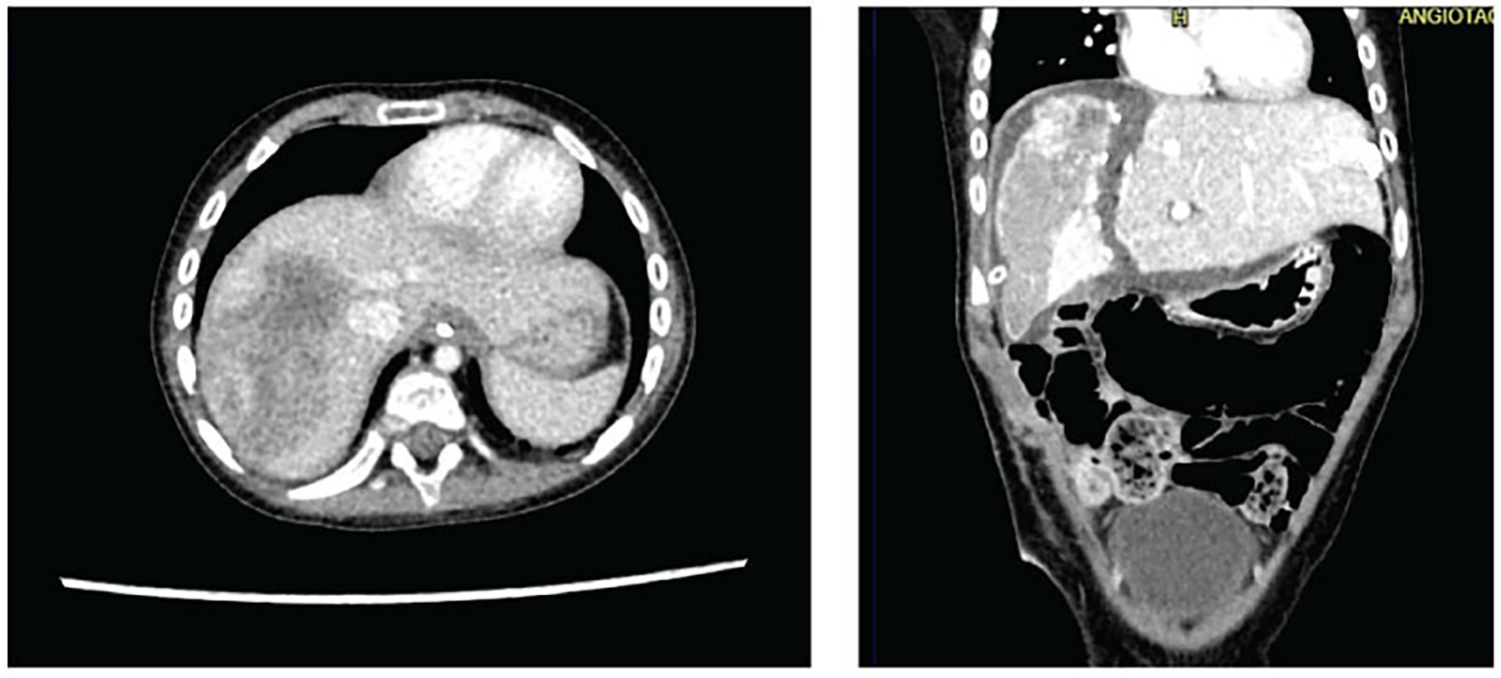
Three-phase liver angiotomography evaluation of Case 2. (Left) Preoperative. (Right) Hypertrophy and surgical changes following stage 1 of ALPPS procedure.

With these results, the case was once again reviewed at a multidisciplinary meeting by the same departments involved initially, and it was decided to proceed with tumor resection employing the ALPPS hepatectomy technique. The surgeries were performed by a general surgery specialist, who has completed fellowships in both pediatric surgery and transplant surgery, and assisted by an additional transplant surgeon. The first surgical stage included ligation of the right portal vein and hepatic parenchymal transection. A follow-up three-phase liver angiotomography was conducted on the 10th postoperative day, revealing a mass in the right hepatic lobe measuring 6.4 × 4 cm (previously 7 × 5 cm) and a volume of the left hepatic lobe measuring 251 cc (Figure 2). The patient underwent the second surgical stage on the 14th postoperative day, during which a complete right hepatectomy was performed. She recovered in the pediatric ICU without complications and was discharged on the 10th day after the second surgical procedure. The pathological report showed a hepatoblastoma with pure epithelial differentiation, specifically 60% embryonal and 40% fetal. The tumor showed a 40% response to chemotherapy, with 60% tumor viability. There was no vascular invasion, however tumor resection margins showed focal tumor involvement in an area of 2 mm.

The patient remained under the care of the pediatric oncology/hematology department. Post-operative treatment was consolidated with chemotherapy following Block C of the SIOPEL 4 protocol. The transition from the SIOPEL 3 to the SIOPEL 4 protocol was based on pathological findings, which revealed tumor involvement within 2 mm of the resection margins. She was readmitted later for the intrahospital administrations of each cycle.

At the 1 year post-surgical follow-up, no apparent abnormalities were detected on physical examination, there was no monthly increase in AFP levels, and there was no evidence of recurrence in the quarterly chest x-ray and abdominal ultrasound.

## 4 Case 3

An 18-month-old female patient, who was under the ambulatory care of a pediatrician, presented with an increase in abdominal circumference over the past 3 months. During the initial evaluation, an abdominal ultrasound showed the presence of a solid mass measuring 52 × 50 mm in the right hepatic lobe. Additional imaging was performed with an abdominal MRI, which revealed a large tumor mass approximately 12 cm in size, involving the left lobe of the liver and possibly segment V, with infiltration into the middle hepatic vein. A chest x-ray showed no evidence of pulmonary metastatic lesions. Based on the PRETEXT classification, it was categorized as PRETEXT III, with an initial alpha-fetoprotein level of 6,130 ng/ml. Neoadjuvant chemotherapy was initiated without a prior biopsy, following the PLADO protocol.

After chemotherapy cycle 3, a follow-up MRI was performed, which reported a lesion in segments II, IV, and V of the right lobe of the liver. This lesion had decreased in size and showed no vascular involvement or other abnormalities. Upon completing the fifth cycle of neoadjuvant chemotherapy, the patient was referred to a higher complexity institution to consider the possibility of a liver transplant. Upon admission, the patient was restaged with a new abdominal MRI that reported a mass in segment IVb, compatible with hepatoblastoma. There was no infiltration of vascular structures or signs of extrahepatic intra-abdominal involvement. A chest CT scan was also performed, which showed no evidence of disease extension. The AFP levels were measured at 447 ng/ml.

The case was reviewed in a multidisciplinary meeting involving the Pediatric Oncology/Hematology, Pediatric Transplant Surgery, and Pediatric Hepatology departments. Feasibility of surgical resection was evaluated with a three-phase liver angiotomography which showed involvement of segment IVb by a solid mass measuring 5.5 × 3.5 × 5 cm, with a patent splenoportal circulation, patent hepatic artery, and patent suprahepatic veins without tumor involvement. Given these findings, it was considered that resection was possible. The total hepatic volume was determined to be 473 cc. The FLR-V, targeting segments VI and VII, was calculated to be 31% of the total volume, amounting to 146 cc. These segments were chosen for FLR-V assessment in preparation for an extended left hepatectomy, involving the anteromedial segments. The rationale behind this surgical approach was the necessity to achieve clear margins for the resection, requiring the selective ligation of the left portal vein branch and the right anterior branch. The preoperative liver function tests showed a minor elevation in AST levels, with no other significant abnormalities.

**Figure 3:**
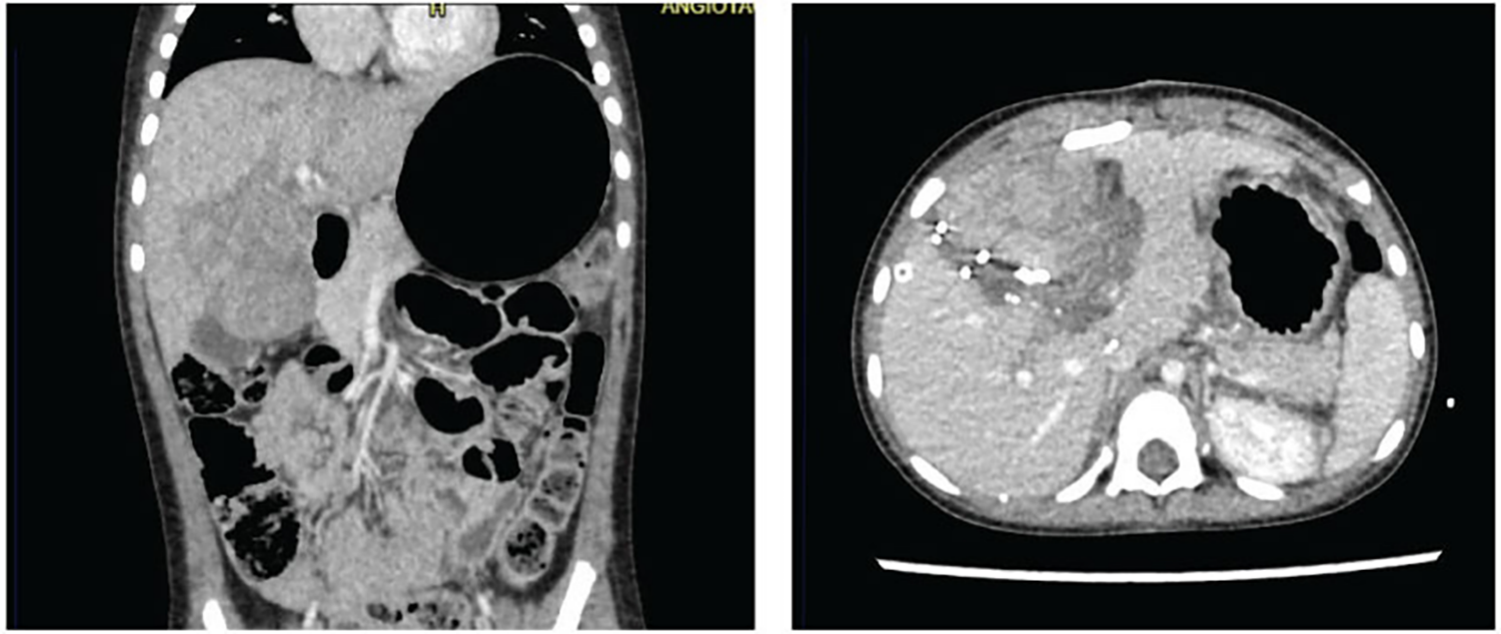
Three-phase liver angiotomography evaluation of Case 3. (Left) Preoperative. (Right) Hypertrophy and surgical changes following stage 1 of ALPPS procedure.

In response to these findings, a subsequent multidisciplinary meeting, involving the departments initially consulted, agreed to proceed with tumor resection using the ALPPS technique. Despite the FLR-V being above typically accepted thresholds, there was a consensus on the heightened risk of liver failure, attributed to the impact of preoperative chemotherapy and the anticipated need for additional resections during surgery. The surgeries were performed by a general surgery specialist, who has completed fellowships in both pediatric surgery and transplant surgery, and assisted by an additional transplant surgeon. During the first surgical stage, selective ligation of the left portal vein and portal branch of the anteromedial segments and hepatic transection to promote hypertrophy of the posterolateral segments was performed. During the postoperative course, the patient developed septic shock due to central catheter-associated bacteremia caused by Pseudomona Putida. On postoperative day 10, a new three-phase hepatic angiotomography was performed, showing the previously known mass without changes in size, changes secondary to portal vein ligation, and a liver volume of 231 ml for segments VI and VII (Figure 3).

The patient underwent the second surgical procedure on the 14th day after the first intervention, and extended hepatectomy including resection of segments I, II, III, IV, V, and VIII performed. Postoperatively, a biliary fistula was documented 24 h after surgery, so the patient underwent a second intervention, and a suture was performed at the transection edge where bile was more impregnated, as an active fistula site could not be identified.

The pathological analysis identified hepatoblastoma characterized by mixed differentiation, with 90% epithelial and 10% mesenchymal components, and revealed no response to prior therapeutic interventions, evidenced by 100% tumor viability. The examination also confirmed the absence of vascular invasion, margins free of malignancy, and unaltered non-tumoral liver parenchyma. In light of these findings, the pediatric oncology/hematology department after consultations with the pediatric hepatology, pediatric gastroenterology, and pediatric transplant surgery departments, recommended the initiation of post-operative chemotherapy according to Block C of the SIOPEL 4 protocol. Subsequently, the patient was discharged 10 days after the second surgical intervention, following the administration of the initial cycle of postoperative chemotherapy in accordance with the SIOPEL 4 protocol.

The patient continued outpatient follow-ups and the end-of-treatment tests, which included a normal echocardiogram, chest CT, and an abdominal MRI, showing no evidence of local recurrences or spread of disease. There was a slight increase in alpha-fetoprotein (AFP) levels prompting further imaging one month later. This time, an abdominal ultrasound and chest x-ray revealed no evidence of local or distant recurrence, and AFP levels progressively decreased thereafter.

**Figure 4:**
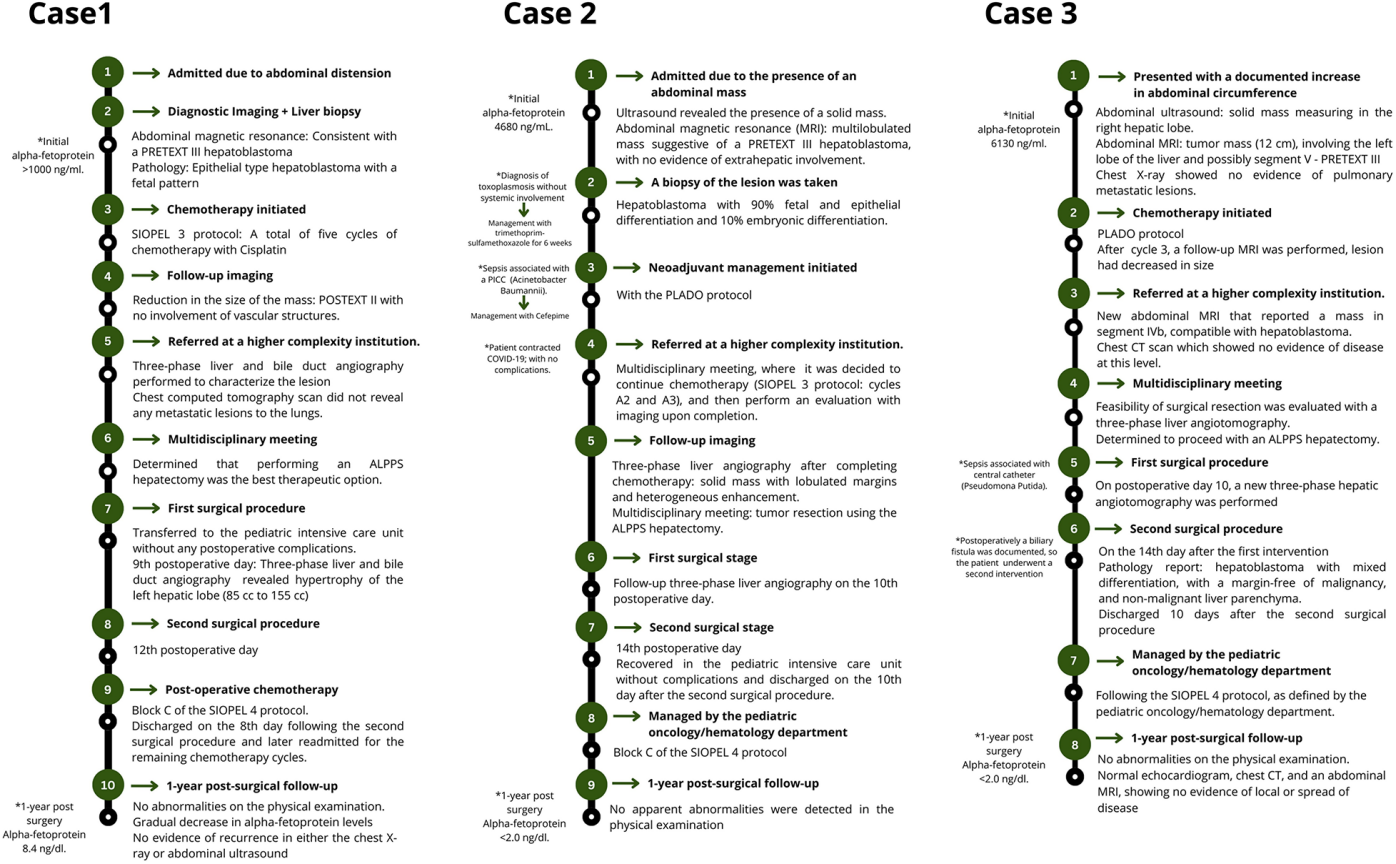
Illustration of the timelines outlining the care provided to all three patients.

At the 1 year postoperative follow-up, no physical exam abnormalities were found, AFP levels did not increase, and no evidence of disease recurrence was evidenced in subsequent imaging (Figure 4).

## 5 Discussion

ALPPS hepatectomy has been proposed as a means of preventing post-hepatectomy liver failure. Its use in pediatric patients with hepatoblastoma is recent and has mainly been described in case series (3, 5–7). Post-hepatectomy liver failure, associated with a high mortality rate of nearly 30% in adult patients undergoing hepatectomies involving four segments (8), places a significant burden on the healthcare system due to the necessity of rescue therapies, including salvage liver transplantation. Therapeutically induced hypertrophy through the ALPPS technique (85%, 132%, and 58% for the three patients, respectively) has enabled the resection of lesions that were previously deemed unresectable, providing a valuable alternative to liver transplantation. However, the absence of comparative data between these therapeutic approaches, limits their general applicability. Patient selection should be conducted on a case-by-case basis, and the availability of liver transplantation should be evaluated in advance.

A central location of hepatoblastomas and their proximity to the portal pedicles and the umbilical fissure often point toward liver transplantation as the only therapeutic option. In a review of liver transplantation experiences in 147 patients with hepatoblastoma, Otte et al. considered PRETEXT IV and PRETEXT III of central location as indications for transplantation (4). While liver transplantation for pediatric malignancies has five-year survival rates of 73% (9), its associated morbidity and costs to the healthcare system make it necessary to explore alternative therapeutic options when feasible. Regarding costs to the healthcare system, Skill et al. found that the costs associated with the surgical procedure and post-transplant follow-up, when compared to resection, were more than twice as high in adult patients with hepatocellular carcinoma (an average of 740,714 USD vs. 316,873 USD) (10). No studies have analyzed costs in pediatric patients with hepatoblastoma, and the applicability of these data is limited by differences in prognosis.

Pertaining to organ availability, Wu et al. reported an average waiting time of approximately 29 days on the transplant list for hepatoblastoma patients in the United States (11); nevertheless, there is decreased availability in developing countries. An analysis of Colombian data spanning from 2009 to 2017 indicated an all-age mean duration on the liver transplant waiting list of 4.6 months, extending up to a maximum of 8 months (12). Within this timeframe, a total of 24 patients succumbed while awaiting transplantation (12). Notably, specific statistics pertaining to hepatoblastoma or the pediatric population remain unreported. Despite the prioritization framework in Colombia, which favors pediatric over adult patients in transplant allocation, the supreme priority is assigned to individuals considered in urgent need of transplantation by the Colombian Instituto Nacional de Salud criteria (13). The selection parameters for urgent transplant status in pediatric patients, although comparatively more flexible than those applied to adults, are infrequently satisfied by hepatoblastoma patients. This regulatory prioritization, while crucial, may potentially result in extended waiting periods for hepatoblastoma patients, thereby delaying their access to transplant surgery.

Patients with a Future Liver Remnant Volume (FLR-V) of less than 25% are most likely to benefit from the ALPPS procedure. This 25% threshold, primarily based on adult patient data, forms the most compelling argument for utilizing the ALPPS procedure in pediatric patients, despite the lack of contrasting information in this group. The procedure has been performed in cases with higher FLR-V; however, without specific pediatric data on acceptable thresholds, such decisions are heavily dependent on clinical expertise and judgment (14). In the scenario of hepatoblastoma patients, the preoperative use of chemotherapy places stress on the liver. Therefore, patients with FLR-V near the accepted thresholds, who have also received preoperative chemotherapy, might be at increased risk of postoperative liver failure (15, 16). This risk influenced the decision to proceed with the ALPPS procedure in case 2. Overall, an FLR-V of less than 35% but greater than 25% may serve as a relative indication for the ALPPS procedure, although decisions must be made on an individual basis. The tumor's location also significantly influences the consideration for the ALPPS procedure. Specifically, central tumors classified as PRETEXT II and PRETEXT III hepatoblastomas could benefit from the procedure due to the necessity for extensive resections. Conversely, PRETEXT II tumors located in segments II, III, VI, and VII, PRETEXT I tumors due to their easily resectable nature, and PRETEXT IV tumors, which necessitate transplantation, are unlikely to benefit from this surgical approach.

The primary benefits of the ALPPS procedure stem from the rapid hypertrophy of the future liver remnant (FLR), which surpasses that of other FLR-enhancing techniques like Portal Vein Embolization (PVE) (17). This accelerated growth increases the chances of achieving complete tumor resections (R0) while potentially reducing the risk of postoperative liver failure, positioning it as a surgical option to consider (17). Additionally, the shortened time span between the two stages of surgery offered by ALPPS minimizes the risk of tumor progression compared to other hypertrophy-inducing methods (17). However, there are associated risks with the ALPPS procedure. The most concerning potential outcome is the rapid growth of the tumor if an R0 resection is not accomplished (2, 17). Yet, this was not observed in any of the cases reviewed. While rapid hypertrophy following ALPPS has not been conclusively proven to enhance liver function directly, the observed reduction in postoperative liver failure rates suggests a positive impact.

Ultimately, the primary limitation for hepatic resections in central location hepatoblastomas, remains insufficient future liver remnants. ALPPS hepatectomy emerges as a viable alternative to liver transplantation for patients with central PRETEXT III hepatoblastoma, especially in situations where liver transplantation availability is limited, and FLR-V are either below or in vicinity of commonly accepted thresholds. Nevertheless, the decision to perform the ALPPS procedure must be made on a case-by-case basis, relying heavily on clinical experience and expertise. Although no tumor recurrences were observed in these cases, rapid tumor recurrences in healthy tissue have been reported with this technique (2). Prospective research studies with larger patient populations are required to assess the incidence of tumor recurrence after this intervention. This will enable a comprehensive comparison of both therapeutic options for hepatoblastomas of all locations.

## Data Availability

The original contributions presented in the study are included in the article/Supplementary Material, further inquiries can be directed to the corresponding author.
